# Quantification of Axial Abnormality Due to Cerebellar Ataxia with Inertial Measurements

**DOI:** 10.3390/s18092791

**Published:** 2018-08-24

**Authors:** Nhan Nguyen, Dung Phan, Pubudu N. Pathirana, Malcolm Horne, Laura Power, David Szmulewicz

**Affiliations:** 1 Networked Sensing and Control Lab, School of Engineering, Deakin University, Waurn Ponds, VIC 3216, Australia; ndn@deakin.edu.au (N.N.); thimydun@deakin.edu.au (D.P.); 2 Florey Institute of Neuroscience and Mental Health, Parkville, VIC 3052, Australia; malcolm.horne@florey.edu.au (M.H.); dsz@me.com (D.S.); 3 Balance Disorders and Ataxia Service, Royal Victorian Eye and Ear Hospital, St Andrews Place, East Melbourne, VIC 3002, Australia; laura_power@live.com.au; 4 Cerebellar Ataxia Clinic, Caufield Hospital, Alfred Health, Caufield, VIC 3162, Australia

**Keywords:** cerebellar ataxia, inertial measurement unit (IMU), postural balance control, rehabilitation, Romberg test, trunk test, entropy measures

## Abstract

Cerebellar Ataxia (CA) leads to deficiencies in muscle movement and lack of coordination that is often manifested as gait and balance disabilities. Conventional CA clinical assessments are subjective, cumbersome and provide less insight into the functional capabilities of patients. This cross-sectional study investigates the use of wearable inertial sensors strategically positioned on the front-chest and upper-back locations during the Romberg and Trunk tests for objective assessment of human postural balance due to CA. The primary aim of this paper is to quantify the performance of postural stability of 34 patients diagnosed with CA and 22 healthy subjects as controls. Several forms of entropy descriptions were considered to uncover characteristics of movements intrinsic to CA. Indeed, correlation with clinical observation is vital in ascertaining the validity of the inertial measurements in addition to capturing unique features of movements not typically observed by the practicing clinician. Both of these aspects form an integral part of the underlying objective assessment scheme. Uncertainty in the velocity contained a significant level of information with respect to truncal instability and, based on an extensive clustering and discrimination analysis, fuzzy entropy was identified as an effective measure in characterising the underlying disability. Front-chest measurements demonstrated a strong correlation with clinical assessments while the upper-back measurements performed better in classifying the two cohorts, inferring that the standard clinical assessments are relatively influenced by the frontal observations. The Romberg test was confirmed to be an effective test of neurological diagnosis as well as a potential candidate for objective assessment resulting in a significant correlation with the clinical assessments. In contrast, the Trunk test is observed to be relatively less informative.

## 1. Introduction

Gait and balance related disabilities are common features among patients with cerebellar ataxia enhancing the possibility of falls leading to possible fractures that would adversely affect their daily life activities [1]. Patients with these neurological conditions often manifest instabilities during regular tasks [2], particularly associated with standing. A number of studies described ataxic postural anomalies and found a strict interrelation between balance deficits and cerebellar ataxia [3]. The complexity of controlling the stability amounts to numerous types of balance perturbations that need systematic clinical appraisal for effective treatment. Although cerebellar ataxic posture is a common and disabling symptom in numerous neurological diseases, systematic studies of the characteristics of cerebellar posture are still to be desired. Moreover, several investigations provide not only conflicting results but also inconsistencies within relevant cohorts [4]. There are currently no specific guidelines to evaluate the clinically relevant changes in postural characteristics. A closer investigation into postural balance control affecting CA patients is likely to enhance traditional clinic-based semi-objective CA assessments.

Postural disabilities in CA are desired to be assessed quantitatively though qualitative [1] assessments are the norm. Typically, the postural assessment in a clinical setting engages the use of subjective rating scales such as the Scale for the Rating and Assessment of Ataxia (SARA). Despite the fact that SARA is validated to detect current progression of CA [5], there is evidence to state that the clinical assessment based rating scales might underestimate the severity of postural changes in CA [6]. Indeed, precise analysis of clinical symptoms is often neglected due to low levels of physician awareness [7]. Therefore, an accurate assessment of postural disorders in CA is pivotal not only in neurological research but also in clinical practice [1].

Traditionally, the Romberg test has become the standard clinical test in the evaluation of postural imbalance during quite standing [8]. In general, this specific test demonstrates loss of stability in the absence of visual input as well as impaired proprioception [9]. When the patient sways or displays an imbalance in a standing posture, with both eyes closed while standing with both feet together, it is considered to be a positive sign for the test. Although this test has been used clinically for many years [10], the sensitivity of this test and precise means of capturing the severity levels of disequilibrium resulting from neurological disability have not been established [11].

In order to assess the postural standing balance for rehabilitation protocols, a number of investigations have developed low-cost telerehabilitation systems allowing continuous monitoring of hemi-paretic stroke patients and their exercises in home-based settings [12,13,14]. Similar studies in the past also mainly employed a range of clinical instruments from force plate platforms to stabilometers for the quantitative assessment of ataxic patients [15]. The use of these systems is still limited in practice due to cost, the specialised technical expertise and the associated laboratories required. Alternatively, objective measures of postural stability using wearable inertial sensors have the potential to assist clinicians with more accurate, stable and sensitive biomarkers for longitudinal testing of posture and gait even during various tasks [16]. Portable, lightweight, and having user-friendly mobile interfaces with automated analysis and real-time display capabilities, the IMU based 3D motion measurement system provides a less expensive, more practical means of quantifying postural sway in both clinical and non-clinical settings [17]. Akira et al. [15] investigated the feasibility of using inertial sensors through various parameters recorded from the back. The findings of this study can be considered sensitive and constitute objective markers for the quantitative clinical assessment of standing and gait impairment in ataxic patients. In [18], trunk body instability was examined using a method based on the power spectral density of an IMU signal from the lower back of the ataxic patient in capturing the truncal sway during quiet stance on a firm surface with eyes opened/closed. Measurements of trunk acceleration during the stance not only determine an impaired postural balance control in individuals with neurological disorders [19], but is also able to track improvement or deterioration in postural stability during the rehabilitation programs [20]. At present, the number of investigations using IMUs still remains small and numerous research questions regarding the type of equipment, optimal placement and the most informative outcome measures are still being investigated due to a lack of detailed studies on how CA affects postural standing and gait [15].

Entropy measures effectively quantify the probability that neighbouring points in a time series will be within a predetermined range, essentially constituting the generic attribute of describing the nature of point-to-point fluctuations in a physiological signal. These information theory based approaches have been considered in the study of postural control to quantify how individuals regulate their postural fluctuations during their daily activities [21]. Nonlinear, non-stationary signals from the force platform are also suited to the relatively localised characterisation and underpin the entropy-based methods by capturing the postural stability to a higher degree of accuracy [22]. Furthermore, there has been a renewed interest in identifying how the *complexity* of physiological signals changes along with the health status [23]. Entropy can also lead to the quantification of changes in physiological movements, possibly due to disabilities such as inability to regulate postural fluctuations [24]. In practice, a wide range of different dynamic models including speech signals and measurements relating to brain and heart signals [25] have been characterised using entropy based approaches. In addition, measures in the entropy domain using force plates or stabilometric platforms for the quantitative evaluation of postural balance control are described in detail through numerous studies [26,27,28]. However, parameters in the entropy domain have never been used before to assess and quantify the upper-body movements measured by a low-cost wearable sensor during quite stance.

Although abnormal oscillations of the trunk are a common clinical feature in patients with cerebellar ataxia, the kinematic behaviour of the upper-body in ataxic patients is still obscure and demands further investigations with closer attention to subtle movements in specific parts of the trunk. Moreover, the role of upper-body movement in ataxic posture is yet unknown [29]. A detailed study of the control of upper-body movements in ataxic patients could be helpful for planning specific rehabilitation treatments or designing new assistive devices aimed at reducing trunk oscillations and improving dynamic stability. In this study, we propose a novel approach to measure and assess the postural balance performance through a clinically used Romberg and Trunk tests by attaching two inertial sensors on two distinct positions: upper-back and front-chest. The captured signal is in the form of time-series acceleration measurements. The entropy of the deduced velocity is primarily considered as neural motor control during a quiet standing posture that contains a significant portion that is proportional to body sway velocity [30]. In fact, it is indicative of a postural control strategy during quiet standing that relies notably on velocity information [31]. Healthy subjects and patients with diagnosed CA disorders are engaged in this study to quantitatively assess postural stability thorough commonly used entropy descriptions against clinical observations.

## 2. Materials

### 2.1. Trial Participants

Thirty-four patients diagnosed with cerebellar ataxia (mean age of 47.64 and a standard deviation of 10.8 years) and twenty-two aged matched healthy subjects were engaged in this investigation. All of the participants performed in both of the bedside tests considered as functional tests for capturing upper-body ataxia movements. This project was approved by the Human Research and Ethics Committee, Royal Victorian Eye and Ear Hospital, East Melbourne, Australia (HREC Reference Number: 11/994H/16). Written consents were obtained from all trial subjects.

### 2.2. Inertial Sensor

**Figure 1:**
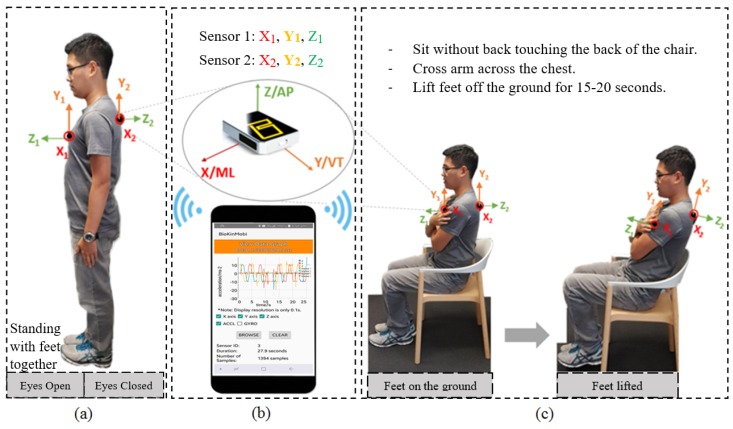
(**a**) Romberg test; (**b**) BioKin${}^{TM}$ system; (**c**) Trunk test.

BioKin${}^{TM}$ is a wireless wearable device with an embedded tri-axial accelerometer [32] (Model chipset “MPU-9150” from InvenSense, Inc., San Jose, CA, USA) connecting with an associated application program on the mobile device as shown in Figure 1b. It is optimised to reduce settling effects and sensor drift problem by eliminating board-level cross-axis alignment errors between each inertial sensor [33]. The sensors were benchmarked against a conventional multiple camera based optical motion tracking system (Vicon system, T40S, Oxford, UK), a high precision benchmarking system [34]. The output data of the sensor was originally sampled at a specific frequency of 50 Hz. Data transmitted through wireless means was processed in MATLAB (R2017b, MathWorks, Natick, MA, USA) environment.

In this research, we engaged two BioKin${}^{TM}$ sensors to add spacial diversity to postural measurements as well as to investigate the feasibility of using an alternative position (e.g., upper-back) of the body that was less likely to interfere with daily activities and had enhanced wearability. This is in contrast to the capture of truncal movement through accelerometer readings in [35]. One sensor unit was positioned on the sternum (front-chest) by means of an elastic neoprene belt [36]. The second sensor was attached on the upper-back location, in the midline and just below the neck as depicted in Figure 1. Indeed, for wearability and patient’s comfort related issues, the aim of these placements was to use a minimal number of sensors as well as use locations that are least likely to have other motion artefacts (i.e., movements of the shoulder, respiration, etc.). These sensors provided trunk acceleration in three orthogonal axes considering as Anterior-Posterior (AP), Medial-Lateral (ML) and potentially Vertical (VT) directions when performing the Romberg and Trunk tests. In fact, these accelerations are measurements of truncal sway. The directional acceleration values were used to deduce the velocity in three axes of movements (*x*, *y* and *z*) defined in the accelerometer’s local coordinate frame.

### 2.3. Signal Processing

**Figure 2:**
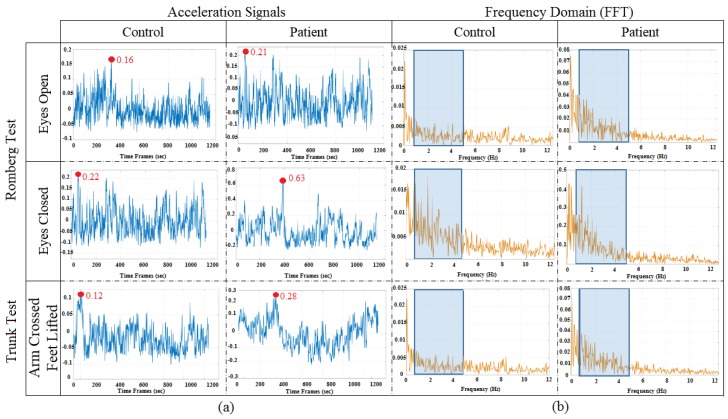
(**a**) Magnitude of the measured accelerations and (**b**) signal frequency bands considered.

All data transmitted through wireless means were processed offline in the MATLAB environment (MATLAB R2017b, MathWorks, Natick, MA, USA). The frequency range of information content is examined with an initial sampling rate set to 50 Hz from the BioKin${}^{TM}$ sensors. As the first sensor is attached on the front-chest using a belt, specific movement synchronised with the respiratory rhythm was also captured. A frequency-domain analysis of the posturographic signal indicates a peak within a relative band between 3 and 5 Hz that is characteristic of cerebellar tremor [1], and the maximum normal respiration frequency for normal persons was considered to be 18 breaths per minutes, which means approximately 0.3 Hz. Furthermore, a low frequency variation of the balance related vertical movement is typically observable in the vertical direction. Figure 2a shows a typical magnitude of the captured acceleration values for each test while Figure 2b denotes the respective band of frequencies used in the underlying analysis. Both of these are removed by the zero-phase Butterworth high-pass filter with the cut-off frequency of 0.3 Hz [37], which essentially contributed to reducing the sensor drift due to the mean amplitude displacement from zero (DC offset) of the steady-state acceleration signals when deducing the velocity. In capturing significant signal energy and removing the unwanted measurement noise, a zero-phase Butterworth low-pass filter with the cut-off frequency of 5 Hz was also employed prior to deriving the velocity.

### 2.4. Clinical Protocol

The experimental setup for the Romberg test and Trunk test as shown in Figure 1b,c is described as follows.

#### 2.4.1. Romberg Test

The subjects were instructed to keep both arms by the sides of the body, with both feet together and maintain this standing posture for 30 s. The assessment was conducted under two different conditions: eyes open (EO) and eyes closed (EC). If the subject exhibited postural instability with a risk of a fall, then the test will be terminated.

#### 2.4.2. Trunk Test

The participants were required to sit without their back touching the back of the chair with arms crossed and positioned against the chest. They were then required to lift their feet from the ground for 15–20 s.

## 3. Methods

### 3.1. Root Mean Square

Root Mean Square (RMS) value of the acceleration has commonly been used in a number of gait and postural analysis studies [38]. Computation of the RMS is extremely simple and requires no preconditions such as an optimal threshold and accurate peak detection to obtain characteristics of the signal pattern [39]. Generally, the magnitude of acceleration is considered in these studies and other signal parameters such as signal peaks. RMS values generally capture the signal effects in a global sense and correlate with the standard deviation since sensor bias in the acceleration signals were removed. The root mean square value of acceleration measurements in orthogonal Cartesian directions *x*, *y* and *z* denoted as $R_{X},R_{Y}$ and $R_{Z}$, respectively, are estimated as $R=\sqrt{\frac{1}{n}\left(acc_{1}^{2}+acc_{2}^{2}+acc_{3}^{2}+\ldots +acc_{n}^{2}\right)}$, where acc represents the time series of acceleration measurements. The RMS value in time series is calculated as follows:

(1) $$RMS=\sqrt{R_{X}^{2}+R_{Y}^{2}+R_{Z}^{2}}.$$

### 3.2. Approximate Entropy (ApEn)

ApEn [40] was derived from the basic concepts of Kolmogorov–Sinai Entropy [41] where signals that encompass meaningful information were subjected to the presence of noise. For an *N* sample time series, $\left\{u(i):1\leq i\leq N\right\}$, given *m*, which generates vector sequences $X_{1}^{m}$ and $X_{N-m+1}^{m}$ as follows:

(2) $$X_{m}^{i}=\left\{u(i),u(i+1),\ldots ,u(i+m-1)\right\},$$

where i,j=1,…,N−m+1 and *m* is the length of the compared window. For each i≤N−m+1, let $C_{i}^{m}\left(r\right)$ be ${\left(N-m+1\right)}^{-1}$ times the number of vectors $X_{1}^{m}$ within the radius of similarity *r* of $X_{1}^{m}$. By defining:

(3) $$\phi^{m}\left(r\right)={\left(N-m+1\right)}^{-1}\sum_{i=1}^{N-m+1}lnC_{i}^{m}\left(r\right),$$

where *ln* is the natural logarithm, Pincus [40] defined the ApEn parameter as follows:

(4) $$ApEn\left(m,r\right)=\lim_{N\to \infty }\left[\phi^{m}\left(r\right)-\phi^{m+1}\left(r\right)\right].$$

For finite *N*, it is estimated by the statistics and hence Equation (4) will become:

(5) $$ApEn\left(m,r,N\right)=\phi^{m}\left(r\right)-\phi^{m+1}\left(r\right).$$

### 3.3. Sample Entropy

In 2000, Richman and Moorman introduced a new measure, Sample Entropy (SampEn). SampEn provides a technical improvement over that of the ApEn algorithm by not counting self-matches and not using a template-wise approach [24]:

(6) $$SampEn\left(m,r,N\right)=-ln\left[\frac{\phi^{m+1}\left(r\right)}{\phi^{m}\left(r\right)}\right],$$

where *m, r, N* and ϕ retain their meanings from Equation (5).

### 3.4. Fuzzy Entropy (FuzzyEn)

According to [42], we can define the parameter FuzzyEn(m,r) of the time series as follows:

(7) $$FuzzyEn\left(m,r\right)=\lim_{N\to \infty }\left[ln\varphi^{m}\left(r\right)-ln\varphi^{m+1}\left(r\right)\right],$$

where $\varphi^{m}\left(r\right)={\left(N-m\right)}^{-1}\sum_{i=1}^{N-m}\phi_{i}^{m}\left(r\right)$ and $\varphi^{m+1}\left(r\right)={\left(N-m\right)}^{-1}\sum_{i=1}^{N-m}\phi_{i}^{m+1}\left(r\right)$.

Additionally, for a specific case of finite datasets, Equation (7) can be estimated by the statistic:

(8) $$FuzzyEn\left(m,r,N\right)=ln\varphi^{m}\left(r\right)-ln\varphi^{m+1}\left(r\right).$$

A systematic review reveals that the selection of two parameters as *m* and *r* are relatively consistent across literature studies: sequence length *m* is typically 2 and point matching tolerance *r* is either 0.15 or 0.20 in order to incorporate in entropy analyses produce statistically reliable and reproducible results results for most data sets [27].

In general, the velocity which is integrated over time from the filtered acceleration, will be considered as the vector time series $X_{i}$ (in Equation (2)) to input to these entropy measures.

### 3.5. Statistical Analysis

In this study, two statistical test parameters were performed; the *p*-value and the area under the ROC curve (Receiver Operating Characteristic) in order to test the efficiency of regularity measures in signal discrimination. The *p*-value represents the probability of indicating samples of one population as equal to or greater than samples of another population and is obtained using a two-sample *t*-test. Numerically, *p* can take values between 0 and 1, and, in this study, *p* < 0.05 was considered as statistical significance. In another aspect, the area under the ROC curve (AUC) is the probability that, for instance, a classifier ranks a randomly chosen instance *x* higher than a randomly chosen instance *y*, where *x* and *y* are samples taken from two independent populations. An AUC value of 0.5 indicates that the distributions of the features are similar in two groups with no discriminatory power. In contrast, an ROC area value of 1.0 would mean that the distribution of the features of the two groups do not overlap at all. The AUC value was approximated numerically using the trapezoidal rules [43] where the larger the ROC area, the better the discriminatory performance. In addition, Spearman’s rank correlation coefficient *r* were calculated to investigate the correlations between the entropy values and the clinical assessment scores.

## 4. Results

### 4.1. Romberg Test

**Figure 3:**
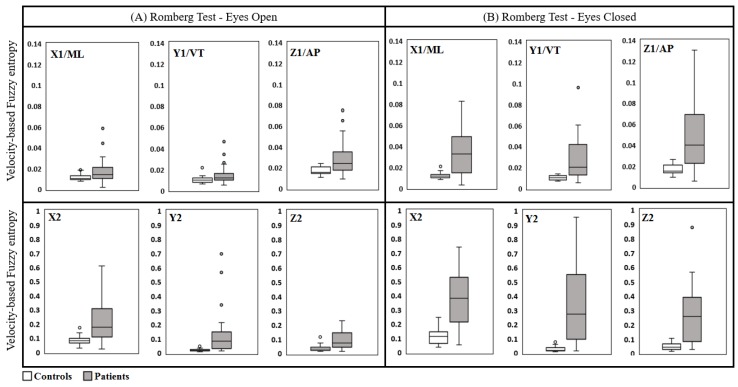
Box-plots of velocity based Fuzzy entropy values in the Romberg test for Sensor 1 (top) and Sensor 2 (bottom); (**A**) is for the eyes open condition and (**B**) is for the eyes closed condition.

The entropy measures of the deduced velocities corresponding to patients were significantly greater than those corresponding to control subjects. This was the case with both eyes open and eyes closed based on trunk acceleration measurements derived from both sensors as depicted in Figure 3. Postural control measured when eyes were closed (EC) had significantly increased for both patients and controls as expected. However, the extent of the difference was conspicuous for the patients when compared to healthy subjects. The level of significance was very high for all parameters measured in the Medial-Lateral and Longitudinal directions. These findings agree with the results obtained using a stabilometer [44], but triaxial accelerometers are more readily employed, particularly for patients with a tenuous balance.

Table 1 presented Spearman’s rank correlation coefficients between the entropy-based features (SampEn, ApEn, FuzzyEn) and RMS from two sensors (Sensor 1 and Sensor 2) and expert clinical scores for the performance in Romberg and Trunk tests. The correlation values between the clinical scores and the Fuzzy entropy-based features from Sensor 1 in Medial-Lateral (ML), Vertical (VT) and Anterior-Posterior (AP) direction when EC were 0.68, 0.69 and 0.76 (*p* < 0.05), respectively. Similarly, for the case of EO, there were strong relationships between the entropy measures and the scores in three directions (ML ($x_{1}$): *r* = 0.63; VT ($y_{1}$): *r* = 0.63; AP ($z_{1}$): *r* = 0.72). It is noticeable that the EC entropy results from Sensor 2 also displayed a strong correlation in three directions (*r* > 0.6). Table 1 outlines that the SampEn technique provided a relatively moderate correlation in three directions (*r* > 0.5) apart from EO (VT ($y_{1}$)) which was 0.48 while those of Sensor 2 were only high in $y_{2}$ (EC: *r* = 0.78 and EO: *r* = 0.62) with *p* < 0.05. Furthermore, in terms of ApEn, the correlation coefficients were low for both Sensor 1 and 2, except for EO and EC in $y_{2}$ (0.59 and 0.65, respectively). This can amount to the inherent differences in the two entropy techniques. Theoretically, ApEn emphasises each sequence as matching itself to avoid the occurrence of the infinite result due to the logarithm of zero in the calculations. This step can cause several biases in ApEn leading to a lack of consistency for the variations relevant to the time series data. Furthermore, as both SampEn and ApEn techniques are sensitive to parameter selection, a slight change in the parameters can consequently result in the variations to be discontinuous. Indeed, the RMS description based features in all three axes for Sensor 1 correlated poorly with expert clinical assessments (*r* < 0.2) as well as relatively poorly correlating between the two sensors (*r* < 0.5).

**Figure 4:**
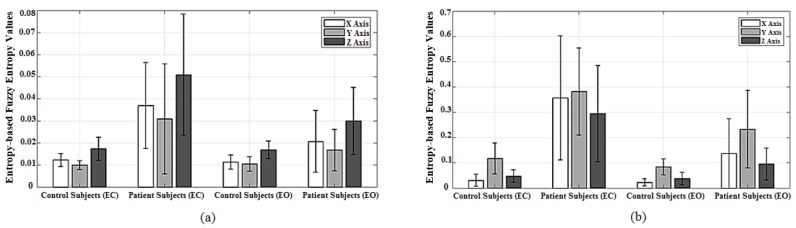
Bar-graphs represent mean and standard deviation values of velocity-based Fuzzy entropy values of Romberg test using two sensors which (**a**) is Sensor 1 and (**b**) is Sensor 2.

The FuzzyEn values, in all three orthogonal directions (ML, VT and AP), were lower for controls in comparison to the corresponding patients values for both EO and EC captured from Sensor 1 as depicted in Figure 4a. In particular, mean ± STD in EC for controls ($x_{1}$: 0.012 ± 0.0029, $y_{1}$: 0.010 ± 0.0021 and $z_{1}$: 0.017 ± 0.0052) were significantly different to the patients’ corresponding values ($x_{1}$: 0.0369 ± 0.0194, $y_{1}$: 0.0308 ± 0.0249 and $z_{1}$: 0.0509 ± 0.0275) in all three directions, respectively. Indeed, this enhanced significant difference (*p* < 0.05) for the case of EC is expected in a strong agreement with the clinical observations of wider acceptance. According to the results derived from Sensor 1 as shown in Figure 4a, FuzzyEn values (mean ± STD) in ML, VT and AP directions were lower for the controls under EC condition ($x_{1}$: 0.012 ± 0.0029, $y_{1}$: 0.010 ± 0.0021 and $z_{1}$: 0.017 ± 0.0052) in comparison to patients ($x_{1}$: 0.0369 ± 0.0194, $y_{1}$: 0.0308 ± 0.0249 and $z_{1}$: 0.0509 ± 0.0275) under similar criterion (*p* < 0.05). Although FuzzyEn was not significantly different within control cohort between EC and EO scenarios, the patients exhibited a more significant difference since EC had a higher mean in entropy values than those for EO in three directions, establishing that the alternate hypothesis holds (*p* < 0.05). In Figure 4b, the means of FuzzyEn values of patients were significantly higher than those of the controls under EC scenario ($y_{2}$: 0.3573 ± 0.2451, $x_{2}$: 0.3821 ± 0.1725 and $z_{2}$: 0.2948 ± 0.1907) and ($y_{2}$: 0.0304 ± 0.024, $x_{2}$: 0.117 ± 0.0611 and $z_{2}$: 0.0482 ± 0.0248), respectively. For the case of EO, the mean values of entropies presented high separation between the two cohorts in all three axes. However, the standard deviation values from Sensor 2 were considerably higher and relatively equal to the mean values, demonstrating a wider range of values for FuzzyEn in comparison to those of Sensor 1.

### 4.2. Trunk Test

A similar behaviour was observed in the Trunk test with a higher level of correlation (Spearman’s correlation coefficient) when using FuzzyEn as shown in Table 1 for both sensors in all three directions in comparison to other techniques. In addition, Sensor 1 demonstrated a stronger correlation rather than Sensor 2 with the expert’s clinical ratings (i.e., $x_{1}$: *r* = 0.53; $y_{1}$: *r* = 0.36; $z_{1}$: *r* = 0.35) (*p* < 0.05) and ($x_{2}$: *r* = 0.41; $y_{2}$: *r* = 0.40; $z_{2}$: *r* = 0.26, respectively). AP ($z_{1}$) movements were well-correlated with clinical assessments when using RMS values with a correlation coefficient of 0.54 (*p* < 0.05) in comparison to the other directional movements (*r* < 0.2, *p* < 0.05).

**Figure 5:**
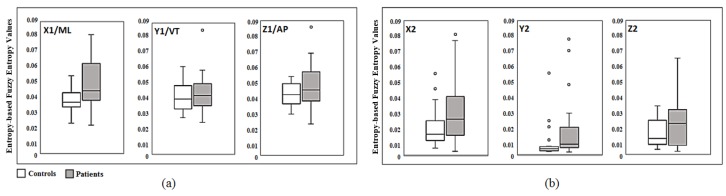
Box-plots of velocity-based Fuzzy entropy results of Trunk test using Sensor 1 (**a**) and Sensor 2 (**b**).

**Figure 6:**
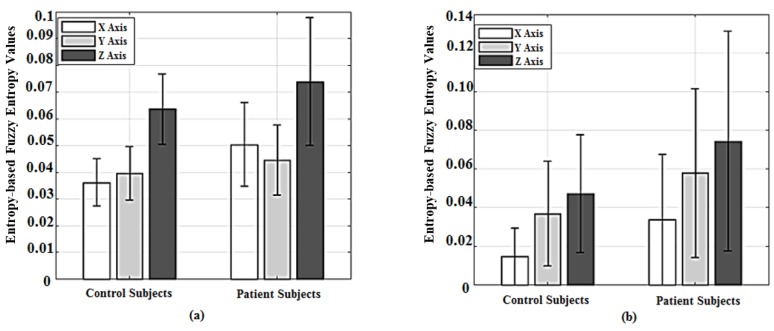
Bar-graphs represent mean and standard deviation values of velocity-based Fuzzy entropy values of Trunk test, whereas (**a**) is Sensor 1 and (**b**) is Sensor 2.

The velocity-based FuzzyEn of patients’ data from Sensor 1 and Sensor 2 presented in Figure 5 were comparatively higher than those of controls demonstrating consistency in terms of complexity of the abnormal cohort across both Romberg and Trunk tests. This can also be observed through the mean values of two cohorts as depicted in Figure 6, where, for the case of Sensor 1, the mean ± STD values of FuzzyEn for controls ($x_{1}$: 0.0368 ± 0.0087; $y_{1}$: 0.0395 ± 0.01; $z_{1}$: 0.0634 ± 0.0123) were slightly lower than that of patients ($x_{1}$: 0.051 ± 0.016; $y_{1}$: 0.0447 ± 0.0139; $z_{1}$: 0.0761 ± 0.0239). Similarly, the mean values of the patients from Sensor 2 were higher than that of the controls ($y_{2}$: 0.0337 ± 0.0307; $x_{2}$: 0.0579 ± 0.0437; $z_{2}$: 0.0744 ± 0.0569) and ($y_{2}$: 0.0146 ± 0.0106; $x_{2}$: 0.0369 ± 0.027; $z_{2}$: 0.0472 ± 0.0303).

## 5. Discussion

Despite the literature reports of abnormal oscillations of the trunk in affected patients [45] and an abnormally increased trunk sway during stance [46], no quantitative studies have yet been performed to provide a detailed analysis of upper-body kinematics in cerebellar ataxia. A closer investigation into subtle postural balance control in CA patients, particularly with respect to directional abnormalities, is likely to enhance traditional clinic-based semi-objective CA assessments.

In this study, the deduced directional velocities were considered as opposed to the measured accelerations in the three orthogonal directions. A number of different entropy approaches were examined to effectively quantify the underlying complexity of the velocity deductions corresponding to two IMUs placed on front-chest (Sensor 1) and upper-back (Sensor 2) positions in order to capture the postural disturbance in participants diagnosed with CA. Sensor measurements facilitated the information of the disability affecting different directions including ML, VT and AP in two distinct locations. In addition, the ability of these postural quality indices to effectively discriminate the two cohorts under separate scenarios (EO and EC) and their correspondence with the commonly used clinical scores were investigated. The proposed approach facilitated the importance of truncal movements to uncover intrinsic disability related information from patients naturally attempting to maintain the postural equilibrium. To the best knowledge of the authors, this is the first preliminary study using entropy measures to analyse the irregularity of velocities derived from trunk accelerations using inertial sensors placed on upper-back and front-chest positions. In this work, we considered the disability manifestation in three orthogonal directions as opposed to most of the literature considering two directions (ML and AP) [44,46] with significant classification observed in the third vertical (VT) direction.

### 5.1. Entropy as a Complexity Measure

Engaging entropy methods has proven to be more sensitive and effective than the traditional Center of Pressure (COP) in capturing the changes of human balance. Entropy based approaches have drawn a significant amount of attention due to their sensitivity in determining the regularity and complexity of the physiological signals [21]. In the entropy domain, the FuzzyEn was introduced to overcome and outperform several existing limitations of other entropy measures such as ApEn and SampEn, whereas both algorithms employ a Heaviside function to measure the similarity of the embedding vectors from the time series being compared [47]. With regard to this advantage, the FuzzyEn approach provided the most significant correlation with the clinical assessments in comparison to other algorithms as shown in Table 1. Therefore, in contrast to the other conventional approaches of using the RMS method [36,37,48], in which Moe-Nilssen reported that the RMS values along the ML direction in a slightly balance-impaired participant increased compared with that in normal subjects [49], our proposed technique can provide a superior quantitative measure of postural balance in order to effectively characterise the disability due to CA.

A significant level of classifications were exhibited between healthy subjects and patients for both Trunk and Romberg tests under each scenario (EC and EO) when using FuzzyEn of the velocity parameters. CA disorder seems to affect the postural balance in all three directions to varying degrees and exhibits more pronounced fluctuations in terms of velocity measure corresponding to the sway movement. Our findings here corroborated with the results reported through previous studies [15]. These have also inferred that the difficulties in controlling truncal stability and reduced anticipatory postural adjustments, likely demarcated by the increased entropy values in all three directions, can indeed result in an increased risk of falls. The significant differences between entropy indexes under EO and EC conditions depicted in Figure 3 revealed a higher degree of truncal fluctuations in all three directions for CA patients, confirming that the closure of eyes consequently results in the exclusion of visual feedback input affecting the postural control. This observation is in agreement with previous studies in which the postural sway was more severe with eyes closed than with eyes open, and the degree of difference was more conspicuous in the patients rather than in the control subjects [15,36]. According to the clinical assessments, the patients are classified into three different sub-groups ranging from 0 to 2, representing the less severity degree (0) to the most severity degree (2), respectively. This classification is attributed to the high variability of entropy values in patients, since higher entropy values imply a higher degree of severity, leading to relatively high STD of Romberg entropy-based measures in patients as shown in Figure 4. Indeed, the fuzzy entropy results are well-correlated to the true patient severities of the underlying condition as depicted in Table 1 in terms of correlation with the clinical scores. Therefore, these outcomes could underline that the changes in entropy domain did truly reflect the impairment in postural balance control due to CA. Considering the results pertaining to fuzzy entropy analysis, CA patients always produced higher values in three directions in comparison to controls in both tests. Indeed, the assertions are in line with the existing literature whereby increases in entropy values are indicative of a physiological signal exhibiting a greater degree of complexity [23].

Interestingly, due to the positional difference of the upper-back sensor, the oblique vertical movement is dominant in the disability manifestation when engaged in the Romberg test (Figure 3) rather than other orthogonal directions. In contrast, for the Trunk test, FuzzyEn results of AP direction are more dominant (Figure 5) in comparison to other axes. Moreover, the front-chest sensor captured the CA related disability more effectively in the form of postural sway, particularly in the AP direction. The abnormalities were prominent in the natural measurement direction (inclined direction) of the upper-back sensor that is unlikely to be observed during normal clinical observations.

### 5.2. Assessment Overview of Romberg and Trunk Tests

The underlying investigation was aimed at (i) the classification of the two cohorts and (ii) achieve a higher degree of correlation with the expert clinical assessments. A strong correlation implies that the proposed technique captures the features that manual examination inherently observes. It is vital that we endeavour to capture features that are unlikely to be captured through clinical observations. Therefore, instances where lesser correlation with a higher degree of classification are also of primary interest to any objective measurement approach.

As shown in Table 2, in terms of discriminatory ability, two sensors agree that the EC scenario was effective in determining the human body sway in comparison to EO. It is noticeable that the upper-back sensor achieved a significantly better classification result, while the front-chest sensor would produce a higher correlation with specialist’s observations. Therefore, it should be noted that the discriminatory capabilities are not always associated with higher correlation with clinical scores implying that certain sensory features may not be directly observable during standard clinical assessments.

Table 1 and Table 2 infer that the upper-back sensor proved to be a potential position for a single sensor setup to classify two cohorts evident through high AUC values, while the front-chest measurements are well-correlated with clinical observations. These findings suggested that designing a wireless wearable system with an upper-back sensor may be a preferred installation for neurologists and clinicians to capture disabilities displayed as postural truncal irregularities. This is an effective and relatively unobtrusive way to provide an objective assessment when making clinical decisions for patients with CA disorder. This is particularly the case when assessments are made in non-clinical environments in order to observe the conditions of the patients in more regular and time intervals at a higher frequency.

An overview of classification and correlation capabilities as depicted in Table 1 and Table 2 shows that the Romberg test appears better suited for capturing the CA disability for the characterisation of postural balance than the outcomes provided by the Trunk test. Consequently, a Romberg test can be considered as a superior and efficient assessment to capture and quantify the abnormality in clinical settings, inferring the redundancy of the Trunk test.

Following the development of low-cost measurement systems in home-based healthcare environments [12,13,14], our proposed approach involving wearable sensors provides additional information of truncal movement in all three orthogonal directions of human movement as opposed to the traditional methods of using 2D trajectories. Indeed, the IMU based system is able to clearly differentiate patients and control subjects even in this simple standing task (EO), which is similar to [15,18,36,50]. These findings are basically in agreement with those obtained using a stabilometer [44]. Therefore, the parameters obtained by the IMUs could provide excellent markers for the objective and quantitative evaluation of postural disturbance in ataxic patients. Moreover, while longitudinal studies are important for greater understanding of postural balance disabilities due to CA, the robust implementation aspect in a wearable platform highlighted in this study can be useful in investigating day-to-day changes of human stability control, particularly in non-clinical settings.

The aforementioned method clearly captured significant differences between CA patients and normal subjects in postural stability, even covering certain clinically unobservable instances. Clinical trials that are generally used to assess an impaired postural stability expressing large variability (e.g., Clinical Test of Sensory Interaction for Balance and others). This disadvantage could be eliminated using the proposed system and method which can be used to evaluate more subtle changes in postural stability in CA patients. As a consequence, the proposed approach may prove useful in gaining deeper insight into the postural stability and postural balance impairments, laying the basis for novel neurological examination and rehabilitation protocols.

### 5.3. Limitations of the Study

Though the results are promising and the inferences are clearly unambiguous, there remain a number of areas where further studies are necessary to confirm the underlying assertions. Larger independent samples of patients and controls, potentially from a range of demographic and geographic areas can validate the consistency of these outcomes. Larger data sets are also essential for further analysis employing knowledge based and data mining approaches to substantiate these underlying characterisations as well as to ensure robust implementation of the underlying inertial sensor based wearable platform in non-clinical settings. The disability traits having potential links to other variations such as gender, age, ethnicity can also be investigated with a larger number of respective cohorts.

## 6. Conclusions

This study is aimed at devising an objective assessment scheme using inertial measurements to capture axial disability manifested due to Cerebellar Ataxia. The determination and assessment of postural instability are proposed to be conducted using the fuzzy entropy of the postural sway velocity deduced from the measured truncal accelerations. Fuzzy entropy descriptions of kinematic features such as velocity were identified as an effective and superior approach in comparison to the RMS based approach considered previously. This technique exhibited significant differences in postural stability control between patients and controls. From the two widely used truncal ataxia tests as Romberg and Trunk tests, the Romberg test was demonstrated to be superior in terms of diagnosis as well as high correlation with the expert clinical assessments under the proposed objective measurement criteria. The Trunk test is observed to be redundant in this context. Furthermore, the positioning of inertial sensors can play a pivotal role in capturing the underlying disability and specifically in obtaining certain movements of interest that are not directly observable during routine clinical assessments. In summary, our results demonstrate a strong argument for wider usage or inertial measurements for the quantitative assessment of cerebellar ataxia, possibly in non-clinical settings. Though clinical scales can represent general trends, the proposed method may prove instrumental in gaining clearer insight into the postural stability and postural balance problem, enhancing the uptake and promoting the use of wearable sensors in longer-term monitoring scenarios facilitated by low-cost measurement systems in non-clinical environments such as home-based healthcare settings under appropriate neuro-rehabilitation programs.

## Figures and Tables

**Table 1:** Spearman’s rank correlation coefficients between Entropy measures and Clinical scores.

Methods	Romberg Test				Trunk Test	
	Sensor 1		Sensor 2		Sensor 1	Sensor 2
	Eyes Open	Eyes Closed	Eyes Open	Eye Closed		
SampEn (ML)	0.5214	0.6719	0.6207	0.777	0.3176	0.2129
SampEn (VT)	0.4835	0.5382	0.1162	0.1373	0.2523	0.0037
SampEn (AP)	0.5069	0.5202	0.2533	0.3877	0.3357	−0.1376
ApEn (ML)	0.2861	0.3475	0.5851	0.6547	0.1454	0.2173
ApEn (VT)	0.1102	0.3826	0.2474	0.1819	0.2606	0.2421
ApEn (AP)	−0.0035	0.1831	0.3789	0.1002	0.0551	0.1687
FuzzyEn (ML)	0.6324	0.7925	0.5969	0.7422	0.5282	0.4098
FuzzyEn (VT)	0.6751	0.6813	0.4936	0.6884	0.3539	0.3956
FuzzyEn (AP)	0.714	0.7581	0.4865	0.6166	0.3485	0.264
RMS (ML)	0.1834	0.0691	−0.2021	−0.6224	0.2015	−0.4593
RMS (VT)	−0.1618	−0.0288	0.3245	0.4358	−0.1329	0.1222
RMS (AP)	−0.1363	−0.1862	−0.1053	0.2701	−0.0786	0.544

**Table 2:** Discrimination evaluation based on area under ROC curve (AUC) values.

Directions	Romberg Test				Trunk Test	
	Sensor 1		Sensor 2		Sensor 1	Sensor 2
	Eyes Open	Eyes Closed	Eyes Open	Eyes Closed		
*x*-Axis	0.7353	0.8035	0.8771	0.9265	0.7031	0.7376
*y*-Axis	0.7126	0.8356	0.8048	0.8812	0.5743	0.6708
*z*-Axis	0.7701	0.8596	0.7901	0.8904	0.6022	0.5921
